# The role of cross-sectional imaging of the extracranial and intracranial vasculature in embolic stroke of undetermined source

**DOI:** 10.3389/fneur.2022.982896

**Published:** 2022-08-26

**Authors:** Hediyeh Baradaran, Hooman Kamel, Ajay Gupta

**Affiliations:** ^1^Department of Radiology and Imaging Sciences, University of Utah, Salt Lake City, UT, United States; ^2^Department of Neurology, Weill Cornell Medicine, New York, NY, United States; ^3^Feil Family Brain and Mind Research Institute, Weill Cornell Medicine, New York, NY, United States; ^4^Department of Radiology, Weill Cornell Medicine, New York, NY, United States

**Keywords:** cerebrovascular disease/stroke, atherosclerosis, carotid artery stenosis, magnetic resonance angiography, carotid artery disease

## Abstract

Despite an extensive workup, nearly one third of ischemic strokes are defined as Embolic Stroke of Undetermined Source (ESUS), indicating that no clear etiologic cause has been identified. Since large vessel atherosclerotic disease is a major cause of ischemic stroke, we focus on imaging of large vessel atherosclerosis to identify further sources of potential emboli which may be contributing to ESUS. For a stroke to be considered ESUS, both the extracranial and intracranial vessels must have <50% stenosis. Given the recent paradigm shift in our understanding of the role of plaque vulnerability in ischemic stroke risk, we evaluate the role of imaging specific high-risk extracranial plaque features in non-stenosing plaque and their potential contributions to ESUS. Further, intracranial vessel-wall MR is another potential tool to identify non-stenosing atherosclerotic plaques which may also contribute to ESUS. In this review, we discuss the role of cross-sectional imaging of the extracranial and intracranial arteries and how imaging may potentially uncover high risk plaque features which may be contributing to ischemic strokes.

## Introduction

Despite an extensive workup, nearly one-third of ischemic strokes are defined as Embolic Stroke of Undetermined Source (ESUS) meaning that no definite cause of the stroke has been identified (1). ESUS has proven to be a difficult clinical entity to treat with an almost 5% per year stroke recurrence rate (1). Most non-lacunar ischemic strokes are embolic and can originate from cardiac sources, from more proximal arterial structures, such as the carotid arteries or aortic arch, or potentially from a venous source in the setting of paradoxical embolism.

Cardiac sources, specifically atrial fibrillation, were thought to play a major role in ESUS because occult atrial fibrillation was found in many patients with ESUS (2). However, two major randomized clinical trials comparing oral anticoagulants to aspirin in patients with ESUS had neutral results (3, 4), suggesting that other embolic causes for ESUS may play a larger role. A major contributor to ischemic stroke is large artery atherosclerotic disease accounting for approximately 25% of ischemic strokes and most commonly arising from the extracranial carotid artery. According to the most common methods for classifying stroke etiologies, in order to attribute an ischemic stroke to large artery atherosclerosis, there must be associated luminal stenosis of at least 50% (5). These criteria do not take into account the recent paradigm shift in our scientific understanding of the contribution of specific plaque features to ischemic stroke.

In this review article, we will review the role of cross-sectional imaging of the carotid arteries in patients presenting with ESUS. First, we will discuss the current standard of care and typical imaging workup to exclude carotid disease as a potential cause of stroke. We will then discuss the role of computed tomography angiography (CTA) and magnetic resonance angiography (MRA) in evaluating potential causes of ischemic stroke in the extracranial carotid artery. We will also review the role of intracranial vessel wall MR (VW-MR) in assessing intracranial atherosclerosis, another potential contributor to ESUS.

## Current paradigm/standard of care

Rather than being a diagnosis of exclusion, ESUS has a standardized, criteria-based definition requiring specific imaging and clinical workup. In order to meet criteria for a diagnosis of ESUS, an ischemic stroke must be a non-lacunar stroke detected on CT or MR imaging, the patient must have ≤ 50% luminal stenosis of the extracranial and intracranial vessels supplying the territory of the brain infarction, and have no major risk of a cardioembolic source or other specific identifiable cause of stroke, such as arteritis, dissection, migraine/vasospasm, or drug misuse. In order to make this diagnosis, suggested diagnostic assessment in evaluating those with ESUS is a brain CT or MR, 12-lead electrocardiogram, precordial echocardiography, cardiac monitoring for 24 h with automated rhythm detection, and imaging of both the extracranial and intracranial arteries supplying the area of brain ischemia with either digital subtraction angiography, MR or CT angiography, or cervical duplex and transcranial Doppler ultrasonography (1). These relatively recent guidelines have allowed for standardization in the identification of those with acute ischemic stroke and have made those with ESUS easier to identify. These more rigid definitions have led to more concentrated effort in mitigating stroke in this population and have paved the way for recent large randomized clinical trials (3, 4).

### Limitations of current imaging techniques

While the current diagnostic criteria for ESUS require assessment of both the extracranial and intracranial arterial structures, the primary focus remains on the degree of luminal stenosis. For decades, the degree of stenosis has been the primary indicator of stroke risk in the extracranial and intracranial arteries. Carotid disease is thought to lead to ischemic stroke by two distinctive, but often synergistic factors: flow-limitation in the setting of stenosis leading to hypoperfusion and artery-to-artery embolism from plaque leading to thromboemboli (6). It is likely that hypoperfusion from flow limitation contributes to cerebral ischemia. Further, impaired perfusion in the setting of flow-limitation may lead to a potentially transient embolic event resulting in an infarct. While flow-limiting stenosis is clearly a risk factor for the development of ischemic stroke, there is mounting evidence that the plaque itself, regardless of the degree of accompanying stenosis is likely a contributor to ischemic strokes *via* artery-to-artery embolism (7).

Recent interest in the plaque components have furthered our understanding of the role of plaque features in contributing to embolic strokes. There is strong histopathologic evidence that plaque may have different features conferring higher risk for an embolic phenomenon. The American Heart Association plaque classification describes a spectrum of plaque with certain features, including intraplaque hemorrhage, lipid-rich necrotic core, and surface defects including fibrous cap rupture which are features indicative of more “vulnerable” plaque that is more likely to rupture and lead to emboli (8, 9). With the current recommendations for imaging, specific carotid plaque features are not always appropriately imaged or may not always be identified or treated as important drivers of ischemic stroke. Despite strong scientific evidence supporting the role of vulnerable plaque features in the development of ischemic stroke, specifically in those with non-stenotic carotid atherosclerosis, these plaque features are not always being routinely assessed using the current guidelines. There is strong evidence that specific plaque vulnerable features in non-stenosing plaque are more commonly seen ipsilateral to infarction in ESUS patients (10). By recognizing the importance of non-stenosing plaque in the extracranial and intracranial vasculature, we may potentially be able to reclassify patients originally thought to have ESUS (11).

## Extracranial carotid plaque

Extracranial internal carotid artery atherosclerosis has traditionally been the most common source of large vessel atherosclerotic disease-causing ischemic stroke. The extracranial carotid arteries are always imaged in the setting of acute ischemic stroke to evaluate for a source. The simplest method for imaging extracranial plaque is by duplex ultrasound (US) where the degree of stenosis based on flow measurements can be assessed. US can also evaluate various plaque features which are known to be higher risk, including echolucent plaque (12). Though US certainly plays a major role in the evaluation of stroke etiology and ESUS (13), we will focus on other cross-sectional imaging modalities which can more accurately assess specific plaque features as well as luminal stenosis for a more complete assessment of stroke risk.

When imaging the extracranial carotid artery in the setting of ischemic stroke, there are two major considerations. First, the degree of luminal stenosis must be assessed using a standardized system, most commonly using North American Symptomatic Carotid Endarterectomy Trial (NASCET) criteria (14). This can be accurately ascertained in any of the imaging modalities currently being utilized for the diagnosis of ESUS. In addition to the degree of luminal stenosis, the components of the plaque must also be assessed, even in those with non-stenosing plaque. All imaging modalities for the extracranial carotid arteries can assess plaque features to a certain degree, though some are more well-suited than others.

### MR imaging

**Figure 1:**
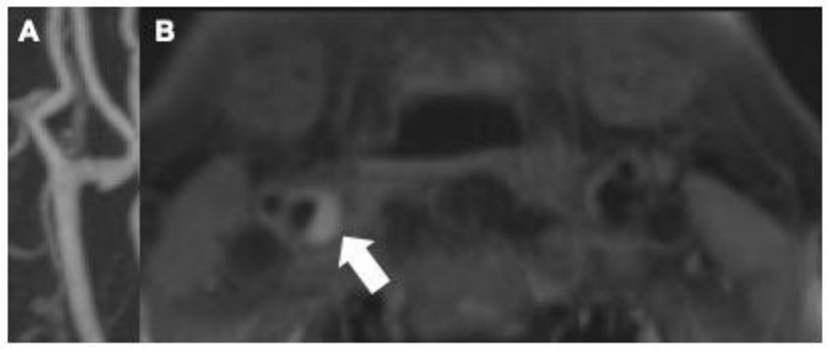
Though there is no accompanying significant stenosis [**(A)** maximum intensity projection of contrast-enhanced MRA], this MPRAGE sequence of the proximal right internal carotid artery in a 73-years-old male demonstrates a large T1 hyperintense plaque [**(B)** arrow]. These findings are compatible with intraplaque hemorrhage, a well-established marker of vulnerable plaque and likely contributor to acute ischemic stroke in this patient.

MR is the most studied method to visualize vulnerable plaque features with considerable evidence supporting its use to evaluate advanced atherosclerotic plaque. Intraplaque hemorrhage (IPH) is the most commonly encountered MR-detected plaque feature considered to be “high-risk” and is strongly associated with infarction (15–17). Other MR-detected vulnerable plaque features including lipid-rich necrotic core, ulceration, or fibrous cap rupture are also strongly associated with ischemic infarction. There is strong histopathologic evidence correlating MR imaging findings to known specific vulnerable plaque features (9, 18). In order to accurately image these plaque features, many utilize dedicated carotid coils or specific high-resolution MR sequences, including T1 and T2 weighted sequences, proton density, and time-of flight sequences to evaluate flow (19). Contrast-enhanced MR sequences can also improve plaque characterization and allow for better characterization of plaque ulceration (20). While using dedicated carotid coils improves imaging by increasing signal-to-noise ratios, some studies have found that even simple MR sequences can accurately identify basic high-risk carotid plaque features (Figure 1) (21).

Multiple studies have specifically evaluated the role of MR imaging of plaque in the setting of ESUS. Several studies have evaluated individuals with ESUS and have found that these MR-detected vulnerable plaque features are more commonly seen ipsilateral to the side of stroke compared to the contralateral side (22–25). Recent prospective studies have confirmed these findings. The Plaque At RISK study showed that in patients with mild-moderate extracranial carotid stenosis, those with IPH and higher total plaque volume were more likely to experience recurrent ipsilateral ischemic stroke over a 5 years follow-up period, though plaque ulcerations and calcifications were not significantly associated (26). Another recent prospective study of patients with non-stenosing plaque found that patients with complicated plaque, such as IPH or surface defects, were much more likely to experience recurrent ipsilateral infarctions in the 30 months following an initial infarct (27). The findings from these studies suggest that even in the setting of non-stenosing plaque, certain higher-risk plaque features may be responsible for infarcts.

Though MR imaging of the extracranial carotid arteries can be helpful in identifying high risk plaque features, its widespread use is somewhat limited by availability, patient contraindications (e.g., implanted metal), and usually lengthy sequences making it a time-consuming imaging examination.

### CTA

**Figure 2:**
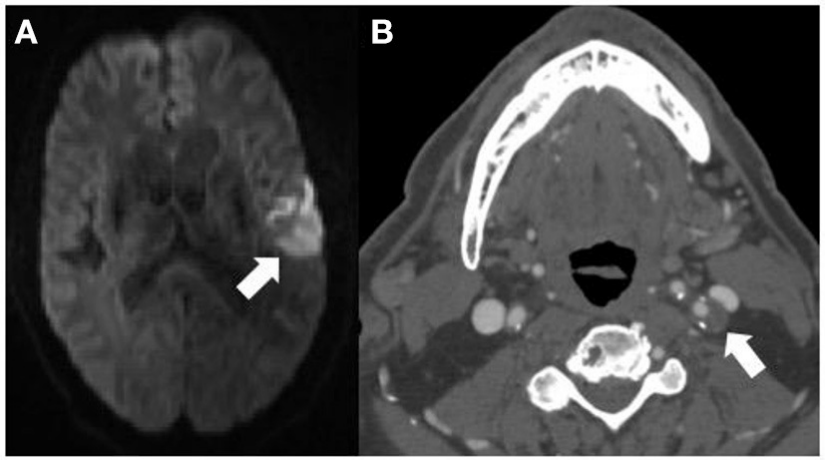
This 71-years-old patient presenting with an acute left middle cerebral artery territory infarction [arrow **(A)**] did not have any significant stenosis by North American Symptomatic Carotid Endarterectomy Trial criteria on CT angiography (CTA) and was thought to have an embolic stroke of undetermined source. The CTA **(B)** does however show a large, predominantly non–calcified plaque up to 5 mm in thickness in the proximal left internal carotid artery [**(B)** arrow] compatible with a vulnerable plaque, potentially the embolic source of the infarction.

CTA is an increasingly commonly used imaging modality in the assessment of etiology of acute ischemic stroke. Because it is relatively cost-effective and quick to obtain, it is most often the first-line examination for those presenting emergently with acute stroke symptoms. While there is more prospective evidence that MR-assessed vulnerable plaque features contribute to future and recurrent ischemic stroke, many of these plaque features can also be assessed using CTA imaging (28). While MR is a superior imaging modality for differentiating histopathologic components of plaque, CTA is able to assess a few specific features which are known to increase risk of emboli, including “soft” or predominantly non–calcified plaque which is thought to be a correlate of IPH or lipid-rich necrotic core, plaque ulceration, and plaque thickness (Figure 2). These features are readily visible on routine CTA imaging and are associated with increased likelihood of symptomaticity (28, 29). Similar to studies performed with MR-detected plaque features, several have found that non-stenotic plaques are more commonly seen ipsilateral to the infarcted cerebral hemisphere in patients with ESUS (30, 31). Specifically, several have found that having plaques >3 mm was more common ipsilateral to the side of stroke (30–32). Other studies have found that plaque with spotty calcification and a “rim sign” were also associated with cerebrovascular ischemic symptoms (33, 34). These studies indicate that though there is a paucity of prospective data evaluating the role of CT-plaque features in future ischemic stroke, certain imaging findings may be useful in identifying those at higher risk of ischemic stroke.

### Other imaging techniques

Though not frequently used in everyday practice, there may be a role for more advanced imaging to evaluate for higher risk plaque. Positron Emission Tomography (PET) imaging has been studied as a method for assessing the vulnerability of carotid plaque. A recent systematic review and meta-analysis found that carotid arteries ipsilateral to recent ischemic events had more avid uptake of markers of inflammatory activity (e.g., 18-F fluorodeoxyglucose) than asymptomatic arteries (35). Other types of more advanced imaging has been studied to evaluate for plaque vulnerability, including dynamic contrast enhanced perfusion imaging (36). These and other findings point to a potential role for advanced imaging in evaluating plaque vulnerability in the future.

## Intracranial atherosclerosis

Intracranial atherosclerosis leads to up to 9–15% of ischemic infarctions in the United States and up to 50% worldwide. Similar to extracranial atherosclerosis, intracranial atherosclerosis must result in at least 50% narrowing in order to be considered causative in the setting of ischemic stroke. Active atherosclerotic plaque can easily be overlooked when using conventional angiographic imaging because plaques do not always produce associated vessel narrowing. Because of this, intracranial vessel wall MR (VW-MR) can be used as an imaging assessment of atherosclerosis, particularly non-stenosing plaque.

### Intracranial VW-MR

Intracranial VW-MR is a powerful tool to image beyond the vessel lumen and for evaluating non-stenosing plaque which may lead to ischemic stroke. In order to accurately assess the vessel wall, there are several critical components to intracranial VW-MR imaging (37). First, in order to highlight the wall itself and any potential plaque, it is essential to suppress flowing luminal blood and CSF, which can be done with a variety of different T1-weighted sequences. This is essential to increase conspicuity of any plaque features or enhancement. Further, high spatial resolution is needed in order to see the small vessel wall with most institutions performing approximately 0.5 mm voxels. Multiplanar acquisitions are also essential to assess the vessels en face because of the inherent tortuosity of the intracranial vessels. This is usually achieved by acquiring images using 3D techniques then creating reformats. Lastly, multiple tissue weightings are also performed to evaluate specific T1 and T2 characteristics in order to distinguish different plaque components.

**Figure 3:**
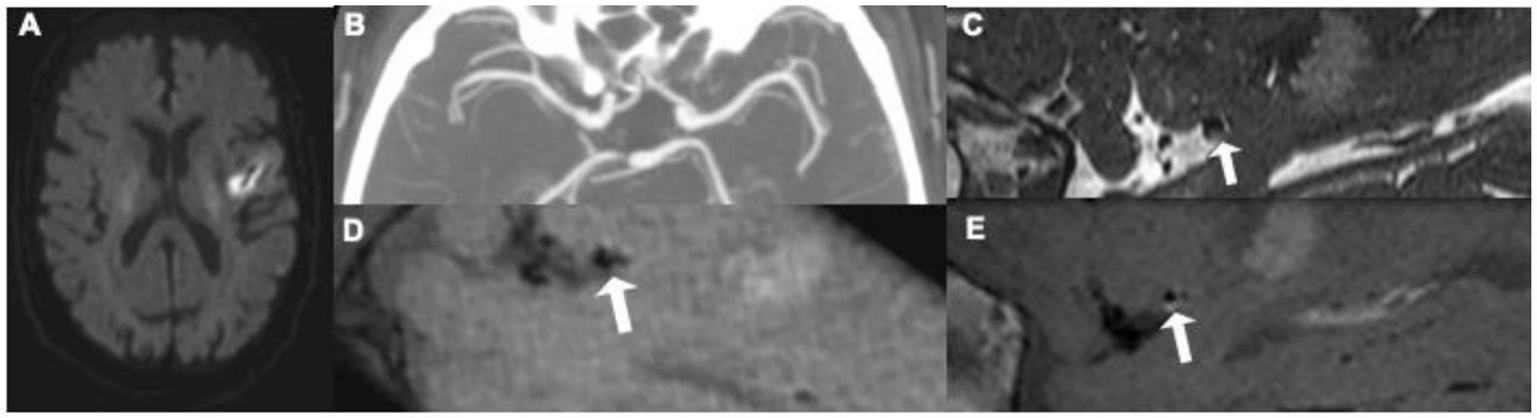
This 62-year old patient presenting with an acute left middle cerebral artery (MCA) infarction on MR **(A)** had CT angiography [**(B)** maximum intensity projection] at presentation without evidence of any significant stenosis. Initially thought to have an embolic stroke of undetermined source, he underwent an intracranial vessel wall MR where he was found to have a focal, eccentric T2 hyperintense [**(C)** white arrow] enhancing [**(D)** pre-contrast image, **(E)** post-contrast image, white arrow] plaque, in the distal M1 segment of the left MCA, thought to be the culprit plaque.4.

Intracranial VW-MR can more easily detect smaller plaques or plaques with associated positive remodeling which may not produce narrowing on angiographic imaging but may still lead to ischemic stroke. Positive remodeling is an adaptive process where the outer wall of a vessel can outwardly bulge in the setting of an atherosclerotic plaque to preserve cerebral blood flow, leading to a normal, non-stenotic appearance on standard angiographic imaging techniques, including CTA, MRA, and DSA. Positive remodeling is commonly seen in the posterior circulation but can be seen in any intracranial arteries. Because of the common occurrence of positive remodeling, many patients presenting with acute ischemic stroke may have a normal appearing angiographic study without any suspicious findings for a contributing atherosclerotic lesion. When imaged using VW-MR, however, culprit atherosclerotic plaques may be identified (Figure 3) and are generally assumed to be causative for ischemic stroke.

VW-MR uses a few specific imaging findings to identify active or culprit atherosclerotic plaques. The most important imaging finding for evaluating plaque in the setting of acute ischemic stroke is plaque enhancement. Several meta-analyses show that plaque/vessel wall enhancement is very strongly associated with culprit or symptomatic plaques (38–40). Plaque enhancement is readily assessable on post-contrast MR sequences and is a fundamental aspect of imaging with VW-MR techniques. In addition to plaque enhancement, positive remodeling is another important imaging finding that is strongly associated with symptomatic plaques (39, 40). This strong association of positive remodeling with symptomatic plaque highlights the importance of VW-MR in identifying potential culprit plaques. Similar to extracranial carotid plaque, discontinuities on the plaque surface indicative of fibrous cap rupture are also associated with ischemic stroke and symptomatic plaques (39, 40). Though intraplaque hemorrhage is a very strong marker of high risk plaque in the extracranial carotid artery, the association between IPH or intraplaque high T1 signal in the intracranial artery is not as strong, with a more modest association with ischemic stroke and symptomatic plaque, more commonly seen in the basilar artery (40, 41). Further studies evaluating the role of intracranial IPH in contributing to acute ischemic stroke are warranted.

When used in patients with ESUS, some studies have found that intracranial VW-MR can be a helpful tool. A study with over 240 patients with ESUS found that intracranial plaque was much more common ipsilateral to the side of stroke (42). They also found that there was increased wall remodeling in patients with ESUS, again highlighting the importance of non-stenosing plaque (42). A recent systematic review of 21 studies of patients with non–stenosing atherosclerosis found that intracranial plaque with higher risk features such as plaque enhancement and positive remodeling were more commonly seen in those with acute infarction, again indicating the role of specific plaque features (43). Another study found that using intracranial VW-MR could change the stroke etiology classification as it identified alternate causes of the ischemic stroke (44).

Intracranial VW-MR has become increasingly popular in evaluating ischemic stroke and ESUS patients, with a recent survey suggesting that more than 50% of neuroradiology practices routinely perform this type of study (45). Despite its increasing popularity, intracranial VW-MR imaging is limited by lengthy acquisitions, patient contraindications, and cost. Further, there has been limited histopathologic validation of MR signal characteristics of intracranial vessel wall pathology due to limitations in correlation with vessel samples (46, 47). This inherent limitation in our ability to correlate imaging findings with histopathologic components constrains our understanding of intracranial plaque characteristics.

## Conclusion

Given recent randomized clinical trial findings that treating cardiac sources for ESUS may not be as beneficial as originally hoped, more attention is being placed on other potential embolic sources. Since the current ESUS definitions require <50% luminal narrowing, potential culprit plaques could be missed or inadequately treated because they are producing insignificant narrowing. In the extracranial carotid artery, both MR and CTA can be used to identify certain plaque features which indicate more plaque vulnerability including IPH on MR and increased soft plaque thickness on CTA. VW-MR can also be used as a powerful tool to identify non-stenosing but active atherosclerotic plaque in the intracranial arteries by identifying an enhancing plaque with positive remodeling. Though these studies can be helpful in determining the source of potential emboli, there are some Further studies are needed to validate these imaging techniques and pave a path for their routine use in ESUS.

## Author contributions

HB, HK, and AG contributed to conception of the manuscript. HB wrote the first draft of the manuscript. HK and AG critically revised and wrote sections of the manuscript. All authors contributed to manuscript revision, read, and approved the submitted version.

## Funding

HB is in part supported by National Institutes of Health grant 5U24NS107156-04. AG is in part supported by National Institutes of Health grants R01HL144541 and R01NS123576. HK serves as a PI for the NIH-funded ARCADIA trial (NINDS U01NS095869).

## Conflict of interest

HK serves as a PI for the NIH-funded ARCADIA trial (NINDS U01NS095869), which receives in-kind study drug from the BMS-Pfizer Alliance for Eliquis^®^ and ancillary study support from Roche Diagnostics; as Deputy Editor for JAMA Neurology; on clinical trial steering/executive committees for Medtronic, Janssen, and Javelin Medical; and on endpoint adjudication committees for AstraZeneca, Novo Nordisk, and Boehringer Ingelheim. He has an ownership interest in TETMedical, Inc. The remaining authors declare that the research was conducted in the absence of any commercial or financial relationships that could be construed as a potential conflict of interest.

## Publisher's note

All claims expressed in this article are solely those of the authors and do not necessarily represent those of their affiliated organizations, or those of the publisher, the editors and the reviewers. Any product that may be evaluated in this article, or claim that may be made by its manufacturer, is not guaranteed or endorsed by the publisher.

## References

[1] Hart RG Diener H-C Coutts SB Easton JD Granger CB O'Donnell MJ. Embolic strokes of undetermined source: the case for a new clinical construct. Lancet Neurol. (2014) 13:429–38. 10.1016/S1474-4422(13)70310-7 24646875

[2] Sanna T Diener H-C Passman RS Di Lazzaro V Bernstein RA Morillo CA. Cryptogenic stroke and underlying atrial fibrillation. N Engl J Med. (2014) 370:2478–86. 10.1056/NEJMoa1313600 24963567

[3] Hart RG Sharma M Mundl H Kasner SE Bangdiwala SI Berkowitz SD. Rivaroxaban for stroke prevention after embolic stroke of undetermined source. N Engl J Med. (2018) 378:2191–201. 10.1056/NEJMoa1802686 29766772

[4] Diener H-C Sacco RL Easton JD Granger CB Bernstein RA Uchiyama S. Dabigatran for prevention of stroke after embolic stroke of undetermined source. N Engl J Med. (2019) 380:1906–17. 10.1056/NEJMoa1813959 31091372

[5] Adams Jr HP Bendixen BH Kappelle LJ Biller J Love BB Gordon DL. Classification of subtype of acute ischemic stroke. Definitions for use in a multicenter clinical trial. TOAST. Trial of Org 10172 in acute stroke treatment. Stroke. (1993) 24:35–41. 10.1161/01.STR.24.1.35 7678184

[6] Caplan LR Hennerici M. Impaired clearance of emboli (washout) is an important link between hypoperfusion, embolism, and ischemic stroke. Arch Neurol. (1998) 55:1475–82. 10.1001/archneur.55.11.1475 9823834

[7] Brinjikji W Huston J Rabinstein AA Kim G-M Lerman A. Lanzino G. Contemporary carotid imaging: from degree of stenosis to plaque vulnerability. J Neurosurg. (2016) 124:27. 10.3171/2015.1.JNS142452 26230478

[8] Stary HC Chandler AB Dinsmore RE Fuster V Glagov S Insull Jr W. A definition of advanced types of atherosclerotic lesions and a histological classification of atherosclerosis: a report from the committee on vascular lesions of the council on arteriosclerosis, american heart association. Circulation. (1995) 92:1355–74. 10.1161/01.CIR.92.5.1355 7648691

[9] Cai J-M Hatsukami TS Ferguson MS Small R Polissar NL Yuan C. Classification of human carotid atherosclerotic lesions with in vivo multicontrast magnetic resonance imaging. Circulation. (2002) 106:1368–73. 10.1161/01.CIR.0000028591.44554.F9 12221054

[10] Kamtchum-Tatuene J Wilman A Saqqur M Shuaib A Jickling GC. Carotid plaque with high-risk features in embolic stroke of undetermined source. Stroke. (20200 51:311–14. 10.1161/STROKEAHA.119.027272 31752616 PMC6993880

[11] Kamel H Navi BB Merkler AE Baradaran H Díaz I Parikh NS. Reclassification of ischemic stroke etiological subtypes on the basis of high-risk nonstenosing carotid plaque. Stroke. (2020) 51:504–10. 10.1161/STROKEAHA.119.027970 31847749 PMC7259428

[12] Gupta A Kesavabhotla K Baradaran H Kamel H Pandya A Giambrone AE. Plaque echolucency and stroke risk in asymptomatic carotid stenosis: a systematic review and meta-analysis. Stroke. (2015) 46:91–7. 10.1161/STROKEAHA.114.006091 25406150 PMC4280234

[13] Komatsu T Iguchi Y Arai A Sakuta K Sakai K Terasawa Y. Large but nonstenotic carotid artery plaque in patients with a history of embolic stroke of undetermined source. Stroke. (2018) 49:3054–56. 10.1161/STROKEAHA.118.022986 30571401

[14] Collaborators^*^ NASCET. Beneficial effect of carotid endarterectomy in symptomatic patients with high-grade carotid stenosis. N Engl J Med. (1991) 325:445–53. 10.1056/NEJM199108153250701 1852179

[15] Gupta A Baradaran H Schweitzer AD Kamel H Pandya A Delgado D. Carotid plaque MRI and stroke risk. Stroke. (2013) 44:3071–77. 10.1161/STROKEAHA.113.002551 23988640

[16] Hosseini AA Kandiyil N MacSweeney ST Altaf N Auer DP. Carotid plaque hemorrhage on magnetic resonance imaging strongly predicts recurrent ischemia and stroke. Ann Neurol. (2013) 73:774–84. 10.1002/ana.23876 23463579 PMC3824333

[17] Saam T Hetterich H Hoffmann V Yuan C Dichgans M Poppert H. Meta-analysis and systematic review of the predictive value of carotid plaque hemorrhage on cerebrovascular events by magnetic resonance imaging. J American Coll Cardiol. (2013) 62:1081–91. 10.1016/j.jacc.2013.06.015 23850912

[18] Yuan C Mitsumori LM Ferguson MS Polissar NL Echelard D Ortiz G. In vivo accuracy of multispectral magnetic resonance imaging for identifying lipid-rich necrotic cores and intraplaque hemorrhage in advanced human carotid plaques. Circulation. (2001) 104:2051–56. 10.1161/hc4201.097839 11673345

[19] Saba L Yuan C Hatsukami T Balu N Qiao Y DeMarco J. Carotid artery wall imaging: perspective and guidelines from the ASNR Vessel Wall Imaging Study Group and expert consensus recommendations of the American Society of Neuroradiology. Am J Neuroradiol. (2018) 39:E9–31. 10.3174/ajnr.A5488 29326139 PMC7410574

[20] Etesami M Hoi Y Steinman D Gujar SK Nidecker A Astor B. Comparison of carotid plaque ulcer detection using contrast-enhanced and time-of-flight MRA techniques. Am J Neuroradiol. (2013) 34:177–84. 10.3174/ajnr.A3132 22627797 PMC7966309

[21] Gupta A Baradaran H Kamel H Mangla A Pandya A Fodera V. Intraplaque high-intensity signal on 3D time-of-flight MR angiography is strongly associated with symptomatic carotid artery stenosis. Am J Neuroradiol. (2014) 35:557–61. 10.3174/ajnr.A3732 24008170 PMC7964710

[22] Freilinger TM Schindler A Schmidt C Grimm J Cyran C Schwarz F. Prevalence of nonstenosing, complicated atherosclerotic plaques in cryptogenic stroke. JACC: Cardiovascul Imag. (2012) 5:397–405. 10.1016/j.jcmg.2012.01.012 22498329

[23] Gupta A Gialdini G Lerario MP Baradaran H Giambrone A Navi BB. Magnetic resonance angiography detection of abnormal carotid artery plaque in patients with cryptogenic stroke. J Am Heart Assoc. (2015) 4:e002012. 10.1161/JAHA.115.002012 26077590 PMC4599540

[24] Gupta A Gialdini G Giambrone AE Lerario MP Baradaran H Navi BB. Association between nonstenosing carotid artery plaque on MR angiography and acute ischemic stroke. JACC: Cardiovascul Imag. (2016) 9:1228–29. 10.1016/j.jcmg.2015.12.004 26897689

[25] Singh N Moody AR Panzov V Gladstone DJ. Carotid intraplaque hemorrhage in patients with embolic stroke of undetermined source. J Stroke Cerebrovascul Dis. (2018) 27:1956–59. 10.1016/j.jstrokecerebrovasdis.2018.02.042 29571754

[26] van Dam-Nolen DH Truijman MT van der Kolk AG Liem MI Schreuder FH Boersma E. Carotid plaque characteristics predict recurrent ischemic stroke and TIA: the ParisK (Plaque At Risk) study. JACC: Cardiovascul Imag. (2022). 10.1016/j.jcmg.2022.04.003. [Epub ahead of print]. 36202450

[27] Kopczak A Schindler A Sepp D Bayer-Karpinska A Malik R Koch ML. Complicated carotid artery plaques and risk of recurrent ischemic stroke or TIA. J Am Coll Cardiol. (2022): 79:2189–99. 10.1016/j.jacc.2022.03.376 35523659

[28] Baradaran H Al-Dasuqi K Knight-Greenfield A Giambrone A Delgado D Ebani E. Association between carotid plaque features on CTA and cerebrovascular ischemia: a systematic review and meta-analysis. Am J Neuroradiol. (2017) 38:2321–6. 10.3174/ajnr.A5436 29074638 PMC7963758

[29] Baradaran H Eisenmenger LB Hinckley PJ. Havenon AHd Stoddard GJ Treiman LS. Optimal carotid plaque features on computed tomography angiography associated with ischemic stroke. J Am Heart Assoc. (2021) 10:e019462. 10.1161/JAHA.120.019462 33586471 PMC8174260

[30] Coutinho JM Derkatch S Potvin AR Tomlinson G Kiehl T-R Silver FL. Nonstenotic carotid plaque on CT angiography in patients with cryptogenic stroke. Neurology. (2016) 87:665–72. 10.1212/WNL.0000000000002978 27412144 PMC4999163

[31] Ospel JM Singh N Marko M Almekhlafi M Dowlatshahi D Puig J. Prevalence of ipsilateral nonstenotic carotid plaques on computed tomography angiography in embolic stroke of undetermined source. Stroke. (2020) 51:1743–49. 10.1161/STROKEAHA.120.029404 32375585

[32] Knight-Greenfield A Nario JJQ Vora A Baradaran H Merkler A Navi BB. Associations between features of nonstenosing carotid plaque on computed tomographic angiography and ischemic stroke subtypes. J Am Heart Assoc. (2019) 8:e014818. 10.1161/JAHA.119.014818 31818209 PMC6951053

[33] Eisenmenger LB Aldred BW Kim S-E Stoddard GJ de Havenon A Treiman GS. Prediction of carotid intraplaque hemorrhage using adventitial calcification and plaque thickness on CTA. Am J Neuroradiol. (2016) 37:1496–503. 10.3174/ajnr.A4765 27102316 PMC7960279

[34] Zhang F Yang L Gan L Fan Z Zhou B Deng Z. Spotty calcium on cervicocerebral computed tomography angiography associates with increased risk of ischemic stroke. Stroke. (2019) 50:859–66. 10.1161/STROKEAHA.118.023273 30879439 PMC6433492

[35] Chaker S Al-Dasuqi K Baradaran H Demetres M Delgado D Nehmeh S. Carotid plaque positron emission tomography imaging and cerebral ischemic disease. Stroke. (2019) 50:2072–79. 10.1161/STROKEAHA.118.023987 31272325 PMC6646056

[36] Yuan J Makris G Patterson A Usman A Das T Priest A. Relationship between carotid plaque surface morphology and perfusion: a 3D DCE-MRI study. MAGMA. (2018) 31:191–9. 10.1007/s10334-017-0621-4 28455630 PMC5813060

[37] Mandell D Mossa-Basha M Qiao Y Hess C Hui F Matouk C. Intracranial vessel wall MRI: principles and expert consensus recommendations of the American society of neuroradiology. Am J Neuroradiol. (2017) 38:218–29. 10.3174/ajnr.A4893 27469212 PMC7963837

[38] Gupta A Baradaran H Al-Dasuqi K Knight-Greenfield A Giambrone AE Delgado D. Gadolinium enhancement in intracranial atherosclerotic plaque and ischemic stroke: a systematic review and meta-analysis. J Am Heart Assoc. (2016) 5:e003816. 10.1161/JAHA.116.003816 27528408 PMC5015301

[39] Lee HN Ryu C-W Yun SJ. Vessel-wall magnetic resonance imaging of intracranial atherosclerotic plaque and ischemic stroke: a systematic review and meta-analysis. Front Neurol. (2018) 9:1032. 10.3389/fneur.2018.01032 30559708 PMC6287366

[40] Song JW Pavlou A Xiao J Kasner SE Fan Z Messé SR. Vessel wall magnetic resonance imaging biomarkers of symptomatic intracranial atherosclerosis: a meta-analysis. Stroke. (2021) 52:193–202. 10.1161/STROKEAHA.120.031480 33370193 PMC7773134

[41] Zhu C Tian X Degnan AJ Shi Z Zhang X Chen L. Clinical significance of intraplaque hemorrhage in low-and high-grade basilar artery stenosis on high-resolution MRI. Am J Neuroradiol. (2018) 39:1286–92. 10.3174/ajnr.A5676 29794236 PMC6039267

[42] Tao L Li X-Q Hou X-W Yang B-Q Xia C Ntaios G. Intracranial atherosclerotic plaque as a potential cause of embolic stroke of undetermined source. J Am Coll Cardiol. (2021) 77:680–91. 10.1016/j.jacc.2020.12.015 33573737

[43] Wang Y Liu X Wu X Degnan AJ Malhotra A Zhu C. Culprit intracranial plaque without substantial stenosis in acute ischemic stroke on vessel wall MRI: a systematic review. Atherosclerosis. (2019) 287:112–21. 10.1016/j.atherosclerosis.2019.06.907 31254918 PMC6707846

[44] Schaafsma JD Rawal S Coutinho JM Rasheedi J Mikulis DJ Jaigobin C. Diagnostic impact of intracranial vessel wall MRI in 205 patients with ischemic stroke or TIA. Am J Neuroradiol. (2019) 40:1701–06. 10.3174/ajnr.A6202 31488500 PMC7028571

[45] Mossa-Basha M Zhu C Yuan C Saba L Saloner DA Edjlali M. Survey of the American society of neuroradiology membership on the use and value of intracranial vessel wall MRI. Am J Neuroradiol. (2022) 43:951–57. 10.3174/ajnr.A7541 35710122 PMC9262066

[46] Turan TN Rumboldt Z Granholm A-C Columbo L Welsh CT Lopes-Virella MF. Intracranial atherosclerosis: correlation between in-vivo 3T high resolution MRI and pathology. Atherosclerosis. (2014) 237:460–63. 10.1016/j.atherosclerosis.2014.10.007 25463074 PMC4262631

[47] Van Der Kolk A Zwanenburg J Denswil N Vink A Spliet W Daemen M. Imaging the intracranial atherosclerotic vessel wall using 7T MRI: initial comparison with histopathology. Am J Neuroradiol. (2015) 36:694–701. 10.3174/ajnr.A4178 25477359 PMC7964300

